# Les cellulites cervico-faciales à propos de 130 cas

**DOI:** 10.11604/pamj.2013.14.88.1477

**Published:** 2013-03-05

**Authors:** Sami Rouadi, Laila Ouaissi, Rhizlane El Khiati, Redallah Abada, Mohamed Mahtar, Mohamed Roubal, Abdellah Janah, Mustapha Essaadi, Fatmi Kadiri

**Affiliations:** 1 Service d’ORL et Chirurgie Maxillo-faciale Hôpital 20 Aout Casablanca, Maroc

**Keywords:** Cellulite cervicale et faciale, étiologie, traitement, Cervicofacial cellulitis, etiology, treatment

## Abstract

Le but de cette étude était d’étudier le profil épidémioclinique et paraclinique de nos patients, d’évaluer leur prise en charge thérapeutique et leur évolution. Nous avons inclus 130 patients pris en charge entre janvier 2007 et novembre 2009. Nous avons relevé de manière rétrospective les données épidémiologiques, les données cliniques, la prise en charge thérapeutique médico-chirurgicale et l’évolution. Notre série retrouve une prédominance masculine avec un âge moyen de 31 ans. L’origine dentaire est l’étiologie la plus fréquente. La tomodensitométrie avec injection de produit de contraste est l’examen clé du bilan initial. L’antibiothérapie et la chirurgie ont permis une bonne évolution dans 74% des cas. Le taux de mortalité est de 0%. Les cellulites cervico-faciales sont des pathologies potentiellement graves touchant souvent des adultes jeunes dont la mortalité hospitalière doit être réduite à la condition d’un diagnostic précoce et une prise en charge médico-chirurgicale immédiate.

## Introduction

Les cellulites cervico-faciales sont des infections des tissus cellulo-adipeux de la tête et du cou. Ce sont des affections graves qui ont une tendance extensive rapide et peuvent mettre en jeu le pronostic vital. Le développement des antibiotiques a permis de modifier radicalement l’évolution de ces cellulites à la condition que leur utilisation soit précoce, adaptée et ne fasse pas oublier le traitement étiologique [1, 2].

## Méthodes

Il s’agit d’une étude rétrospective réalisée au service ORL de l’hôpital 20 Aout 1953 de Casablanca à propos de 130 cas de cellulites cervico-faciales colligés entre janvier 2007 et novembre 2009. Les dossiers étudiés concernaient des patients qui ont nécessité une hospitalisation dans le service. Sont exclus tous les cas traités en ambulatoire ou hospitalisés directement en réanimation vu la gravité du tableau clinique.

## Résultats

Les cellulites cervico-faciales ont représenté 3% de l’ensemble des hospitalisations au service d’ORL avec une moyenne de 47 cas/an.

L’âge moyen de nos patients était de 31 ans avec des extrêmes allant de 2 à 70 ans. Nous avons relevé une prédominance masculine (57%) avec un sex-ratio de 1,3. Vingt pour cent des patients présentaient des antécédents divers répartis comme suit: diabète: 13 cas (10%), tabac: 10 cas (7,7%), hypertension artérielle: 4 cas (3%). Dix-sept pour cent des patients ont reçu une antibiothérapie (9,3%) et/ou un traitement d’anti--inflammatoires non stéroïdiens: (7,7%) avant l’hospitalisation. La porte d’entrée dentaire est la plus fréquente dans 69% des cas, suivie de la porte d’entrée amygdalienne (phlegmon péri-amygdalien) dans 10% des cas puis sinusienne (8%) et cutanée (7%). La porte d’entrée n’a pas été retrouvée dans 6% des cas. Le délai moyen de prise en charge entre le début de la symptomatologie et l’admission au service était de 23 jours avec des extrêmes allant de 1 jour à 180 jours.

**Figure 1:**
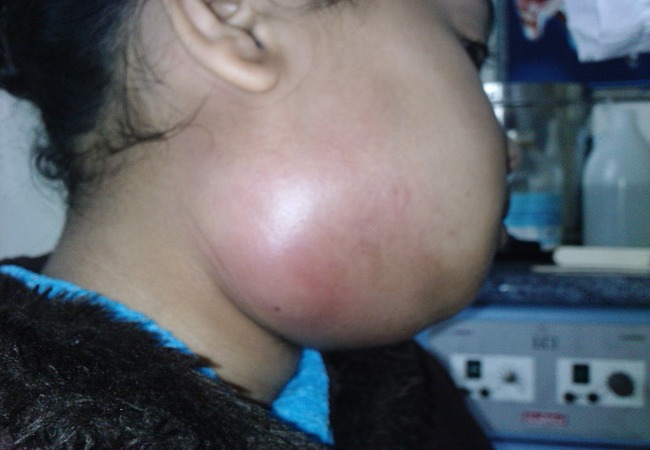
Tuméfaction cervico-faciale inflammatoire

Le signe fonctionnel principal ayant amené les patients à consulter était la tuméfaction cervicale (97%) (Figure 1). Les autres signes fonctionnels comportaient un trismus chez 49 patients (37,6%), une dysphagie dans 5 cas, une dyspnée dans 1 cas et une nécrose cutanée dans 2 cas. Soixante-dix patients étaient fébriles soit 54% des cas et 35 présentaient une altération de l’état général soit 27%. L’examen de la région cervico-faciale a retrouvé dans 54,6% des cas une cellulite périmandibulaire (sous-mentale, submandibulaire), parapharyngée, péri-amygdalienne ou latérocervicale. Dans 23,8% des cas, il s’agissait d’une cellulite périmaxillaire: nasogénienne, buccale ou infratemporale (2 cas).Dans 14,6% des cas on a retrouvé une cellulite orbitaire: préseptale (17 cas) et rétroseptale (2 cas).La cellulite était étendue à toute l’hémiface dans 6,3% des cas. Un seul malade a présenté une cellulite au niveau de la nuque.

Seulement un tiers des patients ont bénéficié d’un prélèvement bactériologique; il s’agissait d’infections polymicrobiennes multi sensibles incriminant des streptocoques et des germes anaérobies.

La tomodensitométrie a été réalisée chez 85% des patients. Elle a objectivé différents aspects: épaississement/infiltration des espaces graisseux (69%), collection abcédée (54%), bulles d’air (10,7%), extension médiastinale (6%), atteinte des parois des axes aéro-digestifs (7,7%).

Tous les malades ont été mis sous antibiothérapie par voie parentérale (Tableau 1). Sept malades ont été mis sous corticoïdes: solumédrol 120mg deux fois par jour. La principale molécule analgésique utilisée était le paracétamol.

**Tableau 1:** Traitement médical instauré en première intention

Protocole ATB	Nombre de patients	Pourcentage (%)
**Amoxicilline-A.clavulanique 4g/j +Gentamycine 160mg/J + Métronidazole 1,5g/j**	74	57
**Ampicilline + sulbactam 4g/j + Amikacine 160mg/j + Métronidazole 1,5g/j**	45	34
C3G(Triaxon*) 2g/j + Gentamycine 160 mg/j + Métronidazole 1,5g/j	6	4,6
Moxifloxacin(Avelox*) 2g/j + Gentamycine 160mg/j + Métronidazole 1,5 G/j	2	1,4

**Figure 2:**
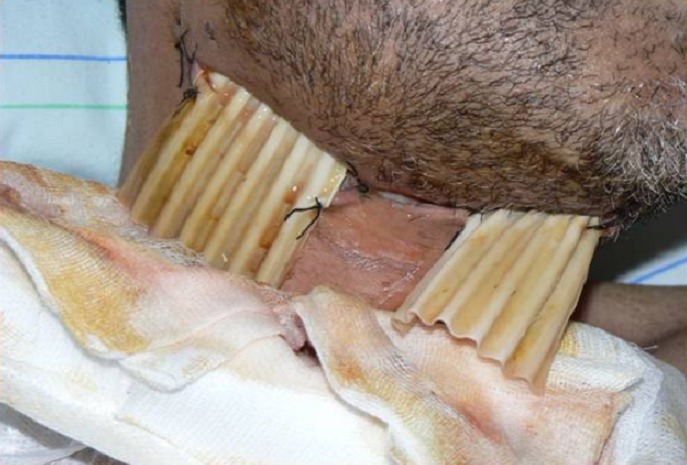
Drainage chirurgical avec mise en place de lames de Delbet

Nous avons pratiqués un drainage chirurgical chez 112 patients (86%) qui a consisté en une incision au bistouri mécanique avec évacuation du pus débridement excision des tissus nécrosés et lavage abondant à la bétadine et à l’eau oxygénée avec mise en place d’une lame de drainage type Delbet chez 6% des patients (Figure 2). Après évacuation du pus, des drainages chirurgicaux et des lavages répétés ont été réalisés. Dix-huit malades (14%) n’ont pas nécessité de traitement chirurgical. L’oxygénothérapie hyperbare n’a été recommandée pour aucun des malades de notre série. Quatre pour cent des patients ont bénéficié d’un examen stomatologique spécialisé.

L’évolution était favorable chez 125 patients (96,3%), le taux de mortalité est nul. Cinq patients ont souffert d’une complication: médiastinite: 4 cas, thrombose de la veine jugulaire interne: 1 cas. Globalement, la durée d’hospitalisation a varié entre 1 et 18 jours avec une moyenne de 4 jours.

## Discussion

Quoique pouvant se voir à tout âge, les cellulites cervico-faciales intéressent le plus souvent l’adulte jeune entre 20 et 30 ans. La prédominance masculine fait presque l’unanimité des auteurs [3–5]. L’origine des cellulites cervico-faciales est le plus souvent dentaire, amygdalienne ou autres (cutanée, sinusienne...). Nos résultats rejoignent ces constatations. L’incidence élevée des cellulites odontogènes est essentiellement due à la mauvaise hygiène bucco-dentaire [6]. Les facteurs favorisants des cellulites cervico-faciales sont nombreux: diabète, intoxication alcoolo-tabagique, immunodépression, prise d’anti-inflammatoires non stéroïdiens [7, 8]. Dans notre série le tabac représente le deuxième facteur de risque retrouvé après le diabète.

Le diagnostic d’une cellulite cervico-faciale est clinique basé sur la conjonction d’un état infectieux grave et de signes physiques cervico-faciaux. La tuméfaction cervico-faciale, quasi constante, est inflammatoire et douloureuse. A elle seule, elle est fort évocatrice de cellulite. L’association à un trismus et à une odynophagie est habituelle. La dyspnée doit faire rechercher une médiastinite surajoutée. Les signes généraux font rarement défaut: fièvre, frissons, sueurs [7, 9].

Les germes en cause sont variables; il s’agit le plus souvent des germes saprophytes de la cavité buccale. La prédominance des germes anaérobies fait l’unanimité des auteurs [10, 11].

La radiographie panoramique dentaire s’impose en cas de cellulite odontogène. Elle peut objectiver les foyers cariés, les zones d’ostéolyse péri-apicale et les foyers granulomateux. La radiographie du thorax fait le diagnostic des complications en particulier la diffusion de l’infection vers le médiastin. La tomodensitométrie grâce à son excellente résolution en densité tissulaire et osseuse permet de préciser la nature inflammatoire d’une tuméfaction cervicale, d’évaluer son extension et de rechercher une collection dont la mise en évidence imposerait le drainage chirurgical. La tomodensitométrie est d’un apport considérable dans le diagnostic d’une extension médiastinale dont les signes initiaux sont parfois extrêmement frustes [12–14].

Les thrombophlébites et les hémorragies peuvent émailler l’évolution des cellulites cervico-faciales [15, 16]. L’extension médiastino-pleuro-péricardique de l’infection est de mauvais pronostic et elle est fréquemment associée à un état septique grave et à une défaillance multiviscérale [17]. Dans notre série, l’extension médiastinale est retrouvée chez 11% des patients.

Le traitement médical est basé sur une antibiothérapie ciblée et efficace rentrant pour les formes graves dans le cadre d’une réanimation adaptée. Les protocoles thérapeutiques sont variables dans la littérature. Pour la plupart, l’association de référence est une trithérapie: Bétalactamines (pénicilline G à la dose de 6 à 20M UI/24h en intraveineuse lente), aminosides qui possèdent un effet synergique efficace sur le staphylocoque et sur certains bacilles gram négatif (Gentamycine à la dose de 160mg/24h) et Métronidazole réputé actif sur les anaérobies à la dose de 1,5g/24h. La dose et la durée du traitement dépendent du type et de l’évolution de la cellulite [18]. Le traitement chirurgical est nécessaire en cas de collections suppurées ou de plages de nécrose. L’intervention doit être la plus complète possible et la voie d’abord large et extensible; il s’agit de drainer mais aussi d’exciser la nécrose et de mettre à plat toutes les zones cellulitiques [19]. En cas de médiastinite associée, un drainage par voie de thoracotomie au cours du même temps opératoire sera envisagé. La trachéotomie s’impose dans les cellulites rétro-pharyngées à cause du risque de rupture de ces abcès lors des manœuvres d’intubation [20]. L’oxygénothérapie hyperbare permet un effet bactériostatique sur les germes anaérobies mais n’est pas d’utilisation courante. Le traitement stomatologique est le plus souvent effectué après refroidissement du processus infectieux. Le meilleur traitement reste préventif: antibioprophylaxie lors des soins dentaires, hygiène buccodentaire, éviction de la prescription abusive d’anti-inflammatoires.

Les différentes séries publiées retrouvent un taux de mortalité de l’ordre de 20 à 40% [21]. Dans notre série la mortalité est nulle; cela est expliqué par le fait que les cas graves ont été hospitalisés d’emblée au service de réanimation.

## Conclusion

Les cellulites cervico-faciales compliquent une infection dentaire ou oropharyngée et se propagent par contigüité vers le cou et le médiastin. La gravité de cette affection nécessite un bilan d’extension des lésions grâce à la tomodensitométrie dont la réalisation ne doit pas différer la mise en route du traitement médico-chirurgical. La mortalité hospitalière des cellulites cervicales doit être réduite à la condition d’un diagnostic précoce et à une prise en charge médicochirurgicale immédiate.
